# Cetuximab, irinotecan and fluorouracile in fiRst-line treatment of immunologically-selected advanced colorectal cancer patients: the CIFRA study protocol

**DOI:** 10.1186/s12885-019-6109-z

**Published:** 2019-09-09

**Authors:** Alessandro Ottaiano, Stefania Scala, Nicola Normanno, Maria Napolitano, Monica Capozzi, Anna Maria Rachiglio, Cristin Roma, Anna Maria Trotta, Crescenzo D’Alterio, Luigi Portella, Carmela Romano, Antonino Cassata, Rossana Casaretti, Lucrezia Silvestro, Anna Nappi, Salvatore Tafuto, Antonio Avallone, Alfonso De Stefano, Mario Tamburini, Carmine Picone, Antonella Petrillo, Francesco Izzo, Raffaele Palaia, Vittorio Albino, Alfonso Amore, Andrea Belli, Ugo Pace, Massimiliano Di Marzo, Paolo Chiodini, Gerardo Botti, Gianfranco De Feo, Paolo Delrio, Guglielmo Nasti

**Affiliations:** 1 Innovative Therapies for Abdominal Metastases Unit, Istituto Nazionale Tumori di Napoli, IRCCS “G. Pascale”, via M. Semmola, 80131 Naples, Italy; 2 Molecular Immunology and Immunoregulation Unit, Istituto Nazionale Tumori di Napoli, IRCCS “G. Pascale”, via M. Semmola, 80131 Naples, Italy; 3 Cell Biology and Biotherapy Unit, Istituto Nazionale Tumori di Napoli, IRCCS “G. Pascale”, via M. Semmola, 80131 Naples, Italy; 4 Abdominal Oncology Unit, Istituto Nazionale Tumori di Napoli, IRCCS “G. Pascale”, via M. Semmola, 80131 Naples, Italy; 5 Scientific Directorate, Istituto Nazionale Tumori di Napoli, IRCCS “G. Pascale”, via M. Semmola, 80131 Naples, Italy; 6 Radiology Unit, Istituto Nazionale Tumori di Napoli, IRCCS “G. Pascale”, via M. Semmola, 80131 Naples, Italy; 7 Hepatobiliary Surgical Oncology Unit, Istituto Nazionale Tumori di Napoli, IRCCS “G. Pascale”, via M. Semmola, 80131 Naples, Italy; 8 Melanoma and Sarcoma Surgery Unit, Istituto Nazionale Tumori di Napoli, IRCCS “G. Pascale”, via M. Semmola, 80131 Naples, Italy; 9 Colorectal Cancer Surgery Unit, Istituto Nazionale Tumori di Napoli, IRCCS “G. Pascale”, via M. Semmola, 80131 Naples, Italy; 10 Medical Statistics Unit, University of Campania, Luigi Vanvitelli, Naples, Italy

**Keywords:** Colorectal Cancer, Antibody-dependent cell-mediated cytotoxicity, Cetuximab, Irinotecan, Fluorouracule, FcγR, Phase II study

## Abstract

**Background:**

Combination of chemotherapies (fluoropirimidines, oxaliplatin and irinotecan) with biologic drugs (bevacizumab, panitumumab, cetuximab) have improved clinical responses and survival of metastatic colorectal cancer (mCRC). However, patients’ selection thorough the identification of predictive factors still represent a challange. Cetuximab (Erbitux®), a chimeric monoclonal antibody binding to the Epidermal Growth Factor Receptor (EGFR), belongs to the Immunoglobulins (Ig) grade 1 subclass able to elicite both in vitro and in vivo the Antibody-Dependent Cell-mediated Cytotoxicity (ADCC). ADCC is the cytotoxic killing of antibody-coated target cells by immunologic effectors. The effector cells express a receptor for the Fc portion of these antibodies (FcγR); genetic polymorphisms of FcγR modify the binding affinity with the Fc of IgG1. Interestingly, the high-affinity FcγRIIIa V/V is associated with increased ADCC in vitro and in vivo*.* Thus, ADCC could partially account for cetuximab activity.

**Methods/design:**

CIFRA is a single arm, open-label, phase II study assessing the activity of cetuximab in combination with irinotecan and fluorouracile in FcγRIIIa V/V patients with KRAS, NRAS, BRAF wild type mCRC. The study is designed with a two-stage Simon model based on a hypothetical higher response rate (+ 10%) of FcγRIIIa V/V patients as compared to previous trials (about 60%) assuming ADCC as one of the possible mechanisms of cetuximab action. The test power is 95%, the alpha value of the I-type error is 5%. With these assumptions the sample for passing the first stage is 14 patients with > 6 responses and the final sample is 34 patients with > 18 responses to draw positive conclusions. Secondary objectives include toxicity, responses’ duration, progression-free and overall survival. Furthermore, an associated translational study will assess the patients’ cetuximab-mediated ADCC and characterize the tumor microenvironment.

**Discussion:**

The CIFRA study will determine whether ADCC contributes to cetuximab activity in mCRC patients selected on an innovative immunological screening. Data from the translational study will support results’ interpretation as well as provide new insights in host-tumor interactions and cetuximab activity.

**Trial registration:**

The CIFRA trial (version 0.0, June 21, 2018) has been registered into the NIH-US National Library of Medicine, ClinicalTrials.gov database with the identifier number (NCT03874062).

## Background

Colorectal carcinoma is a highly incident neoplasm in Western countries with more than 200,000 new cases diagnosed each year in Europe. About 30 % of patients presents with a metastatic disease [1, 2]. In the last 10 years, significant progress has been made in the treatment of metastatic colorectal cancer (mCRC) due to the the introduction of chemotherapy (CT) containing oxaliplatin and irinotecan. The addition of each of the two chemotherapeutics to fluoropyrimidines has increased the objective response rates and improved the overall survival. In addition, the combination of polyCT and new biological drugs (bevacizumab, cetuximab and panitumumab), has doubled the median survival of mCRC patients [3].

Cetuximab (Erbitux®) is a chimeric monoclonal antibody belonging to the Immunoglobulins (Ig) grade 1 subclass [4]. It blocks the binding of the endogenous ligands of EGFR (Epidermal Growth Factor Receptor), thus inhibiting receptor function. EGFR-dependent signal transduction pathways are involved in the control of proliferation, cell survival, angiogenesis and cell migration [5]. Cetuximab binds to EGFR with an affinity that is 5 to 10 times higher than that of endogenous ligands. The drug is indicated for the treatment of patients with mCRC with non-mutated (wild-type) RAS (RAt Sarcoma) oncogene both in combination with CT and monotherapy in patients who have failed oxaliplatin and irinotecan. The mutation of the RAS gene makes it constitutively activated and therefore not susceptible to EGFR inhibition. In mCRC, the incidence of mutations in the RAS gene is between 30 and 50% [6]. Recent evidence shows that patients with non-mutated RAS mCRC have a significantly greater chance of responding to cetuximab or a combination of cetuximab and CT [7].

### Rationale for evaluating FcγR polymorphisms in mCRC

One of the mechanisms of action of cetuximab is the stimulation of ADCC (Antibody-Dependent Cell-mediated Cytotoxicity). ADCC is mediated by immunoglobulins that bind to cellular targets and makes them sensitive to recognition and destruction by immunologic effectors (Natural Killer, macrophages, myeloid-derived cells, etc.) [8]. The effector cells, particularly the Natural Killers (NK), express receptors for the Fc portion of these antibodies (FcγR). The binding affinity between FcγR and Fc portion of the immunoglobulins is critical for the target cell recognition and the extent of the immunologic response [9, 10]. In the general population, genetic polymorphisms of FcγR have been described to modify the binding affinity of the IgG1 Fc fragment [10]. The polymorphisms identified for FcγRIIa (or CD32, predominantly expressed on macrophages) and FcγRIIIa (or CD16, expressed on NK cells and macrophages) are histidine (H)/arginine (R) at position 131 and valine (V)/phenylalanine (F) at position 158, respectively. In recent years, in vitro [11, 12] and in vivo studies [13] have demonstrated that cetuximab induces an NK cells-mediated ADCC against colon cancer cells independently of KRAS (Kirsten RAt Sarcoma) status. Conversely, clinical studies have shown that FcγRIIa-131H/H and FcγRIIIa-158 V/V genotypes (simplified here as FcγRIIa H/H and FcγRIIIa V/V) are associated with improved response and efficacy in follicular lymphomas and metastatic breast carcinomas treated with rituximab and trastuzumab [14–16], respectively. Contrasting results have been reported in mCRC treated with cetuximab [17, 18]. The variability of these results may depend on methodological issues such as the absence of NRAS (Neuroblastoma-Rat Sarcoma) and BRAF (B- Rapidly Accelerated Fibrosarcoma) oncogene mutations’ assessment, populations’ heterogeneity or the lack of characterization of the tumor microenvironment (TM) with particular emphasis to a subset of CD163+ macrophages (M2 macrophages) producing an array of anti-ADCC molecules (e.g. pro-angiogenic and immunosuppressive factors) [19]. We previously showed that the FcγRIIIa V/V genotype (high affinity Fcγ receptor) correlates with better clinical response and improved PFS (Progression Free Survival) in patients with mCRC treated with cetuximab [20, 21], as described by Bibeau et al. [18].

### Previous results of folfiri plus cetuximab in first-line treatment of mCRC

Cetuximab is active and well tolerated in the first-line treatment of mCRC in association with fluoropyrimidine and irinotecan as demonstrated in the randomized, phase III, EMR 62202013 trial [22] in which the combination of cetuximab and irinotecan plus 5-fluorouracile infusion/folinic acid (FU/AF) was compared to CT only. The proportion of non-mutated KRAS patients was 64%. The response rate (RR: complete plus partial responses/number of evaluable patients) was 46.9% (CI: 42.9–51.0) in the CT/cetuximab arm, significantly higher (*p* = 0.0038) than that of the CT arm (38.7%; IC: 34.8–42.8). Odds ratios of subgroup analyses showed that, in the KRAS wt population, the RR of the cetuximab arm was significantly higher (59.3% vs 43.2%, odds ratio 1.91, 95% CI: 1.24–2.93). Furthermore, the association was able to improve PFS with a HR of 0.85 (IC: 0.726–0.998, *p* = 0.0479). The most common grade 3–4 toxicities in the combination arm were diarrhea (15.7% CT plus cetuximab vs 10.5% CT, *p* = 0.008) and those related to cetuximab infusion (2.5% CT plus cetuximab vs. 0% CT, *p* < 0.001). In the phase III, FIRE-3 study, mCRC patients carrying KRAS wt were randomized to folfiri plus cetuximab or folfiri plus bevacizumab first-line treatment [23]. The RR in the cetuximab group was 62.0% (95% CI: 56.2–67.5) and the median PFS was 10.0 months (95% CI, 8.8–10.8). Safety profile was not different from that previously described, with the most common grade 3–4 toxicities being haematologic (25%), skin reactions (26%), and diarrhoea (11%). The proportion of patients achieving an objective response did not significantly differ between the two groups.

### The current therapeutic context of mCRC and CIFRA hypothesis

Recent meta-analyses [24, 25] have evidenced the role of colon tumor side (rigt vs left) as a predictive factor for response to therapy [26]. Accordingly, the RAS wt tumors deriving from the left colon would be more responder to CT in association with anti-EGFR drugs as compared to the right-sided neoplasms. However, the treatment of the RAS wild-type mCRC is currently based on the use of CT doublets (fluoropyrimidine and oxaliplatin or irinotecan) and biological drugs (bevacizumab, panitumumab, cetuximab). This concept is well expressed in the ESMO (European School of Medical Oncology) guidelines [27] in relation to the diversity of the initial treatment intent, suggesting the use of CT doublets and anti-EGFR (panitumumab or cetuximab) when the main objective is a rapid tumor shrinkage. The NCCN guidelines (National Comprehensive Cancer Network v3.2018) contemplate the use of CT and anti-EGFR, panitumumab or cetuximab, in first or second line therapies in mCRC patients whose primary tumor is localized to the left colon. At present, however, due to a lack of sequence studies with high cross-over rates between biologic drugs, it is not possible to state the best biological drug to be used in first-line.

Here we propose the administration of folfiri (fluorouracile and irinotecan) plus cetuximab in patients selected on the FcγRIIIa V/V genotype. In summary, the CIFRA study is set on a hypothetical higher response rate of FcγRIIIa V/V patients than those reported in previous trials (about 60%) and assumes that ADCC is one of the possible mechanisms of cetuximab action. Furthermore, in this immunologically selected cohort, the different responses associated to tumor side (right vs left) could be non existent or attenuated. CIFRA study results could contribute to ameliorate patients’ selection and definitively address the ADCC and FcγR polymorphisms role in cetuximab activity.

## Methods and design

CIFRA is a single arm, single center, open-label, phase II study, assessing the activity of cetuximab in combination with folfiri in FcγRIIIa V/V patients with KRAS, NRAS, BRAF wild type mCRC. It will be conducted at the academic hospital Istituto Nazionale Tumori di Napoli, IRCCS “G. Pascale” in Naples (Italy). The study includes also biomarkers’ analysis (cetuximab-mediated ADCC, TM characterization).

### Objectives

The primary objective is to evaluate the role of the molecular and immunologic selection of patients on response to folfiri and cetuximab in patients with mCRC. The response will be evaluated according to Response Evaluation Criteria In Solid Tumors (RECIST) criteria, version 1.1 [28].

Secondary objectives are the safety, the responses’ duration, the progression-free (PFS) and overall survival (OS). Toxicity will be graded according to the Common Terminology Criteria for Adverse Events (CTCAE) of the National Cancer Institute, version 4.0, June 14, 2010. Response duration will be measured from the time of documented objective response (CR or PR) until documented tumor progression (see also “Response and toxicity assessment” section). PFS will be determined from the data of treatment start untill progression (defined according to RECIST), OS untill death from any cause. Tertiary and correlative objectives consist in exploratory studies of biological markers as predictors of outcome (see the “Tranlsational research” section).

### Ethical considerations

All the procedures described in this protocol have been designed according to the principles of the Good Clinical Practice guidelines of the International Conference on Harmonization (ICH) and of the Declaration of Helsinki. The study was approved by the Ethical Committee of the National Cancer Institute of Naples, Italy (No. 60/18). All patients will provide a written informed consent to CIFRA study clinicians before recruitment and tissue and blood samples collection. In order to protect the privacy of patients included in the CIFRA study, the Structure that has the responsibility for registration, collection and management of personal data, will not provide patient names to persons not involved in the study, with the exception of the Ministry of Health or Ethics Committees (as required by the current legislation only for inspection and control purposes). After registration, a unique and progressive numerical code will be assigned to the patients and shown in the header of all electronic data collection systems and it will be used for all communications regarding the patients. A list of patients’ codes will exist exclusively at the Secretariat of the CIFRA study.

### Study design and statistical analyses

Sample size is based on a two-stage study design by Simon [29] with activity as primary end-point.

The null hypothesis of a not relevant response rate (40%) will be tested against an alternative response rate hypothesis of 70% with a one-tailed test. The test power is 95%, the alpha value of the I-type error is 5%. With these assumptions the sample for passing the first stage is 14 patients with > 6 objective responses. The final sample is 34 patients with > 18 objective responses to draw positive conclusions.

The populations considered for the analysis are described as follows. Safety Population (SP) is defined as all patients receiving at least one dose of treatment. Incorrect treatment or anticipation of the end of treatment are not reasons for exclusion from the SP. Efficacy Evaluable (EE) population is represented by patients who will receive at least one post-baseline assessment of the primary endpoint [28].

All patients who will receive at least one dose of treatment will be included in the descriptive statistics. The basic characteristics of the recruited patients will be reported for the categorical variables as total number and percentage and for the continuous variables as mean and standard deviation or median and interquartile range. Descriptive statistics of patients excluded from the SP and EE will also be carried out. Summary statistics on treatment will be compiled including information on dose changes, interruptions, non-compliance, reasons for protocol deviation, and treatment duration. The analysis on safety will be carried out on the SP. All the information necessary to identify the occurrence of adverse events will be used in the analysis and summarized through descriptive statistics. The confidence intervals will be calculated at 95%. Time-to-outcome will be described by Kaplan-Meier curves.

### Eligibility criteria

The main inclusion criteria are: cytological or histological diagnosis of colorectal adenocarcinoma; KRAS, NRAS, BRAF wild-type; FcγRIIIa V/V genotype; stage IV; age < 75 years; at least 1 measurable lesion; ECOG Performance Status 0 or 1; life expectancy > 3 months; negative pregnancy test for all potentially childbearing women; written informed consent. The main exclusion criteria are as follows: previous systemic anti-tumor treatment (allowed treatment with capecitabine or fluorouracil and radiotherapy in the neoadjuvant setting of rectal tumors with therapy terminated at least 6 months before); presence of primary non-treated stenosing colorectal neoplasm; neutrophils < 2000/mm^3^ or platelets < 100.000/mm^3^ or hemoglobin < 9 g/dl; serum creatinine level > 1.5 times the maximum normal value; GOT and/or GPT > 5 times the maximum normal value and/or bilirubin level > 3 times the maximum normal value; previous malignant neoplasms (excluding basal or spinocellular cutaneous carcinoma or in situ carcinoma of the uterine cervix); active or uncontrolled infections; other concomitant uncontrolled diseases or conditions contraindicating the study drugs at clinician evaluation; presence of brain metastases; refusal or inability to provide informed consent; impossibility to guarantee follow-up.

### Therapeutic schedule

**Fig. 1:**
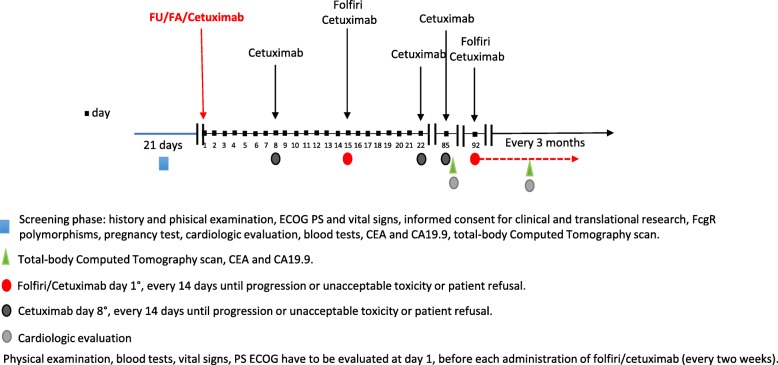
Ideal timeline of CIFRA study

The therapeutic schedule [22, 23] is represented by cetuximab 400 mg/mq intravenously (iv) with a loading dose of 400 mg/mq at the first cycle followed by 250 mg/mq iv weekly. Cetuximab will be diluted in 500 ml of saline solution and administered by iv infusion in 90 min. The administration of irinotecan will precede that of cetuximab and will consist on a dose of 180 mg/mq diluted in 500 ml of saline solution and administered iv in 60 min. Fluorouracil (5-FU) will be administered at a dose of 400 mg/mq in slow iv bolus at half of lederfolin 200 mg/mq (diluted in 250 ml of saline solution) 2-h infusion. An elastomeric pump with 5-fu 2400 mg/mq in continuous 46 h-infusion will be adminstered after lederfolin. The therapeutic scheme just described is briefly indicated as folfiri/cetuximab. Only at the first administration of CT, irinotecan will not be administered (Fig. 1). This is a peculiar and innovative characteristic of the CIFRA study. ADCC occurs rapidly at the beginning of the therapy because it involves lympocytes subpopulatons (i.e. NK) that do not require immunological priming. Although immunomodulatory effects of irinotecan are not described, it is reasonable and biologically plausible that irinotecan-induced lymphopenia could interfere the ADCC phenomena.

Atropine 0.25 mg sub cutis will be administered for the prevention of acute cholinergic events before irinotecan infusion. Premedication with *antihistamines at standard doses and* dexamethasone 4 mg will be applied before cetuximab infusion.

### Duration of therapy

Administration of folfiri/cetuximab will be allowed until progression or unacceptable toxicity. In case of unacceptable toxicity related to irinotecan the patients will continue cetuximab and 5-FU until progression. In case of unacceptable toxicity related to cetuximab the patient will continue folfiri until progression. In case of unacceptable toxicity related to 5-FU the patients will continue with cetuximab and irinotecan until progression.

### Dose modifications for folfiri

Dose reductions wil be applied in relation to the more severe side effect. Once modified, the dose of the drug will be the same for the subsequent cycles. A 20% reduction of folfiri will be permitted in case of grade ≥ 3 of haematologic or non-haematologic toxicities (except for alopecia). Folfiri will be rechallenged after *recovery* of toxicity to baseline or *grade 1* or less. At the second appearance of grade ≥ 3 side effects a dose reduction of 50% will be applied; after a further grade ≥ 3 toxicity or after the first appearance of grade ≥ 2 cardio-vascular chemotherapy will be permanently discontinued. Chemotherapy will also be interrupted in case of postponement of the administration for more than 4 weeks. No prophylactic use of G-CSF o erythropoetin is planned.

### Dose modifications for cetuximab

Cetuximab infusion will be delayed in case of grade > 3 cutaneous toxicity for 2 weeks. The investigator will also consider the administration of topical and/or oral antibiotic drugs and/or topical corticosteroids [30]. The treatment will be reintroduced if the cutaneous toxicity reduces to grade 2 or lower. If grade 3 skin toxicity reoccurs at a second or third time, cetuximab will be postponed for a further 2 weeks, but when reintroduced the dose will be reduced to 200 mg/mq and 150 mg/mq, respectively. Dose reductions of cetuximab are permanent and it will be permanently discontinued in case of 4 weeks suspension or fourth grade 3 toxicity event occurrence despite appropriate dose adjustments. Allergic reactions may occur during or after administration of cetuximab. In case of allergic reaction, the patient will be treated according to clinical practice. The patient should also be asked to report immediately to the doctor if any late reaction occurs. In case of grade 1 allergic reaction the cetuximab infusion rate will be reduced at 50%; it will not be increased subsequently, but rather reduced at any subsequent administration. In case of a second allergic or hypersensitive reaction despite the reduced infusion rate, the infusion will be interrupted and the patient will continue with folfiri alone. If the patient experiences an allergic or hypersensitive reaction of grade > 3, cetuximab will be permanently discontinued and chemotherapy will be continued if considered appropriate by the investigator.

### Timing of exams and procedures

#### Screening phase

The screening phase of the study will begin after signing of the informed consent and it will consist on the evaluation of the inclusion and exclusion criteria and in the collection of a venous blood sample (10 ml) for the determination of the FcγR polymorphisms. Baseline visit and exams will be carried out within 21 days before therapy start (clinical history, clinical examination, PS ECOG, vital signs) along with the beyond described parameters. Blood count and clinical biochemistry will be performed at our local laboratories. The following variables will be evaluated: hemoglobin, blood count with leukocyte formula, platelets, total bilirurbin, AST, ALT, alkaline phosphatase, serum creatinine, total proteins, sodium, potassium, calcium, urea, lactic dehydrogenase, creatinine clearance, CEA and CA19.9. ECG and cardiac ultrasonography with evaluation of the ventricular ejection fraction will be also performed. Pregnancy test will be done for all potentially childbearing women and must be negative during the screening phase. The test can also be repeated during treatment if requested by the local Ethics Committee. All patients, throughout the duration of the study, must use barrier methods for anti-conception. Total-body Computed Tomography with iv contrast or, if contraindicated, Magnetic Resonance (MRI) abdomen and chest computed tomography without iv contrast will be obtained.

#### Treatment phase

Clinical examination, evaluation of vital signs, blood count and clinical biochemistry will be performed at day 1 of each cycle before administration of folfiri/cetuximab (every 2 weeks). Cardiological evaluation, CEA, CA19.9 and assessment of response to treatment will be performed after 3 months of therapy start and thereafter every 3 months until progression.

#### End-of-treatment phase

The suspension of the treatment can occur in case of toxicity, progression, and/or at the request of the patient. Additional treatment lines or follow-up procedures will be applied at the discretion of the clinician responsible of the medical treatment. The final visit will be made immediately after disease progression. The following data will be collected: ECOG Performance Status, clinical examination, vital signs, blood count and clinical biochemistry. The end of study visit should be done within 30 days of the last administration of folfiri/cetuximab and before starting any second-line CT. The cutaneous toxicity, if present, will be followed-up until resolution; if second-line CT will be undertaken the treatment will not start before its resolution. In any case, the outcome of adverse events will be recorded after the the end of study visit. The “patient death form” must be completed when the patient dies. The end-of-study form should be completed for patients who die before the end of study visit and if the patient withdraws consent, preferably before 28–32 days after the last folfiri/cetuximab administration.

### Response and toxicity assessment

The assessment of response will be perfomed through total-body computed tomography with iv contrast (if contraindicated: abdomen MRI and chest computed tomography without iv contrast), CEA and CA19.9 after 3 months of folfiri/cetuximab and every 3 months thereafter until progression. Responses will be classified according to the RECIST v. 1.1. An independent blinded central review of radiologic examinations will be performed by a DMC (Data Monitoring Committee). The independent DMC will be also responsible of reviewing the PFS data. Toxicity will be assessed in accordance with the CTCAE, version 4.0. The CTCAE contains 26 categories of adverse events, organized for pathophysiology, anatomy and etiology. Each adverse event is graded with a scale ranging from 0 (absence of event or value within the normal range) to 5 (death caused by the adverse event). Patients will be assessed for the presence of adverse events at each study visit and will also notify the investigator by telephone in case of significant adverse events reported between one visit and the next. For each adverse event, the maximum grade per patient will be reported. If a patient experiences a toxic effect of any grade on multiple occasions, the event will be counted only once.

### Translational research

Biomarkers will be evaluated in specimens and correlated with outcomes and therapy effectiveness. In particular, the primary tissues, and metastases when available, will be characterized for the presence of tumor-infiltrating M2 macrophages by immunohistochemistry through the expression of CD163 (ab182422, Abcam), TGF-β (ab92486, Abcam), Arginase-1 (GTX113131, Genetex), SSP1 Osteopontin antibody (ab218237, Abcam], and PD-L1 (E1L3N®, XP®). M1 infiltrating macrophages will be detected through the following antibodies: CD86 (ab53004, Abcam), iNOS (ab115819, Abcam), IFN-γ (ab218426, Abcam), TNF (ab1793, Abcam). Natural Killer (NK) and Cytotoxic T Lymphocites (CTL) will be characterized as follows: NKP46+ (Clone 195,314, R&D system), granzymeB (ab134933, Abcam), Foxp3+ (ab20034, Abcam). For FcγR polymorphisms detection, DNA will be extracted from the whole blood lymphomonocyte component of each patient (10 ml of venous blood) by Ficoll-Paque density gradient. The analysis of the V158F and H131R alleles will involve a gene amplification phase by PCR reaction with oligonucleotides specific for the regions of interest and a subsequent PCR products analysis by automatic sequencing (Big Dye terminators version 3.1 cycle sequencing kit and 3130 Genetic Analyzer; Applied Biosystems) [20, 21]. As an internal quality control, samples with previously known sequenced genotype will be used. These investigations will be conducted in the laboratories of the “Cellular biology and biotherapies Unit” of the National Cancer Institute of Naples, IRCCS “G. Pascale”.

To study cetuximab-mediated ADCC, peripheral blood mononuclear cells (PBMC) from patients will be isolated at diagnosis by Ficoll-Paque Plus gradient centrifugation (GE Healthcare), then they will be cultured in complete RPMI-1640 medium enriched with human IL2 (10 ng/mL) for 18 h in order to generate Lymphokine-Activated Killer (LAK) cells. Target CRC cells (HT29) will be plated in a 96-well plate at 1 × 10^4^ cells/well. Twenty-four hours later, LAK will be added at a 10:1 effector:target (E:T) ratio in fresh medium, in the presence of cetuximab (10 μg/mL), or the rituximab (anti-CD20, 10 μg/mL), as negative control, or in the presence of staphylococcal enterotoxin B (SEB) as positive control. Cytotoxicity will be evaluated by sulforhodamine B (SRB) assay [31]. ADCC elicited by not activated freshly prepared PBMCs will be also evaluated at E:T cell concentration ratios of 20:1 and 10:1. The specific cytolysis percentage will be calculated using the following formula: Cytotoxicity (%) = [1 − (mean test optical density/mean optical density target)] × 100 [32, 33]. Cetuximab-mediated ADCC is given by cytotoxicity_with cetuximab_ − cytotoxicity_without cetuximab_. All experiments will be performed in triplicate, and results expressed as mean values ± standard error. These investigations will be conducted in the laboratories of the “Functional Genomics” of the National Cancer Institute of Naples, IRCCS “G. Pascale”.

## Discussion

The identification of predictive biomarkers characterizing the ideal treatment of mCRC patients is subject of intense investigation. RAS oncogenes in mCRC have not completely satisfied this unmet need since about 40% of patients with RAS wt mCRC dose not respond to anti-EGFR treatments. We previously reported in a series of 74 KRAS wt mCRC patients, genotypic frequencies of FcγRIIIa and FcγRIIa of 36% VV, 54% VF, 10% FF and 36% HH, 56% HR, 8% RR, respectively; interestingly, FcγRIIIa but not FcγRIIa polymorphisms were significantly associated with response to anti-EGFR-based therapy [20]. FcγRIIIa polymorphisms had also significant prognostic value for PFS [FcγRIIIa: median PFS 18.2 months in V/V patients (18 patients, 13 events) vs 17.3 months in V/F patients (26 patients, 25 events) vs 9.4 months in F/F patients (5 patients, 5 events); Log Rank test: *p* = 0.04] at univariate and multivariate analyses (HR: 2.35; CI: 1.37–4.01; *p* = 0.001) with grading and response to first-line chemotherapy. In another study, we described genotipic frequencies of FcγRIIa and IIIa in 96 consecutive mCRC patients and 148 control subjects and we analyzed the clinical impact of cetuximab-mediated ADCC in vitro from LAK patients-derived [21]. There were no statistically significant differences between the control and study groups in terms of genotypic frequencies. The incidence of FcγRIIIa V/V, V/F and F/F were: 28, 47 and 23%, respectively. Interstingly, the FcγRIIIa V/V and V/F genotypes were associated with higher cetuximab-mediated ADCC compared with F/F, median 27% (range: 0–76%) and 20% (0–57%) versus 9% (0–36%), respectively (*p* = 0.001). The response rate was higher in patients with the FcγRIIIa V allele (*V/V and V/F* genotypes) compared with the F/F genotype (*p* = 0.025) as well as the PFS of patients with FcγRIIIa V/V and V/F was significantly longer than that of patients with F/F (10.8 vs. 5.1 months respectively, *p* = 0.05, Log Rank test). The extent of in vitro cetuximab-mediated ADCC was significantly correlated with cetuximab clinical response (30% in responders vs 8% in non-responders patients, *p* = 0.020; ANOVA test). These observations prompted us to prospectively design the CIFRA trial, which is an investigator initiated phase II study to verify a superior activity of folfiri/cetuximab of 70% (+ 10% of that previously described) in mCRC patients selected on a genetic polymorphism of FcγRIIIa. The risk of a possible unexpected detrimental effect in this selected population is reduced in CIFRA study by the two-stage design that will stop the enrollment early if the first condition of acceptable activity is not met.

Notably, the CIFRA study will also prospectively address whether the ADCC phenomenon contributes to cetuximab activity. The hypothesis of ADCC as additional or alternative anti-cancer mechanism is supported by two mirror observations: i) a small percent of KRAS mutated mCRC patients respond to cetuximab [34, 35] and, ii) not all “all RAS” wt mCRC patients respond to anti-EGFR therapy [35]. Thus, blockade of signal transduction may not be the only mechanism of action resulting in the clinical benefit of cetuximab [36]. In fact, ADCC induced by EGFR-specific mAbs may inhibit tumor progression in vivo, even in cancers resistant to EGFR signaling inhibition [37]. Immune mechanisms besides molecular alterations, could contribute to cetuximab activity as suggested also by indirect data of Seo and colleagues [38] who reported a significant correlation between EGFR expression and ADCC activity, but not with the mutational status of RAS and BRAF. To this regard, CIFRA results could prompt a clinical pilot study to test the activity of cetuximab-based therapy in RAS mutated FcγRIIIa V/V mCRC patients. Additionally, CIFRA may suggest a more complex evaluation including FcγRIIIa polymorphisms and prompt phase III studies in order to compare panitumumab versus cetuximab with folfiri in RAS wt/FcγRIIIa V/V mCRC patients.

Furthermore, TM characterization (i.e. NK cells, M1 vs M2 macrophages) and cetuximab-mediated ADCC will help to understand any relation between specific signaling pathways and treatment response, as recently reported [39, 40]. Although the description of complex TM biology or dynamics is beyond the scope of this article, herein TM will be evaluated with particular regard to tumor-associated macrophages (TAM), the most represented tumor-infiltrating host cells (may represent up to 50% of the tumor mass). TAM are classically distinct in activated (M1) macrophages and alternatively activated (M2) macrophages [41]. M2 TAMs can produce growth factors and cytokines such as CCL2, CXCL12, CXCR4, TGFβ, VEGF, PDGF, COX-2 and metalloproteinases that determine immunesuppression and stimulate local tumor growth as well as the metastatic process [42–44]. NK cells-mediated ADCC is reduced by M2 TAMs through TGFβ release and induction of a CD27^low^CD11b^high^ NK phenotype [45]. Conversly, M1 TAMs stimulate anti-tumor CD4^+^ and CD8^+^ T cells response and also interact with NK cells that produce IFN-γ to amplify anti-tumor activity [46, 47]. However, the role of TAMs during tumor progression and response to anti-cancer therapies is still under investigation. Interestingly, TAMs express both FcγRIIa and IIIA, but the differential role of M1 vs M2 during administration of therapeutic antibodies remains undetermined. Data from TAM characterization could help to interpret CIFRA results as well as provide new insights in TM interactions and cetuximab activity. In selected patients, cetuximab could be managed as an “immunotherapeutic drug” and association with NK-activating cytokines (IFNs, IL-2) [48] could also gain relevance in the attempt to activate ADCC against antibody-coated cancer cells.

## Data Availability

Not applicable.

## Abbreviations

ADCC: Antibody-Dependent Cell-activated Cytotoxicity
BRAF: B-Rapidly Accelerated Fibrosarcoma
CCL2: C-C Motif Chemokine Ligand 2
CD: Cluster of Differentiation
COX-2: Cyclooxygenase-2
CT: ChemoTherapy
CTCAE: Common Terminology Criteria for Adverse Events
CTL: Cytotoxic T Lymphocites
CXCL12: C-X-C Motif Chemokine Ligand 12
CXCR4: C-X-C chemokine receptor type *4*
ECOG: Eastern Cooperative Oncology Group
EGFR: Epidermal Growth Factor Receptor
ESMO: European School of Medical Oncology
F: Phenylalanine
FA: Folinic Acid
FcγR: Fragment-cc γ Receptor
FU or 5-FU: 5-FluoroUracile
G-CSF: Granulocyte-Colony Stimulating Factor
H: Histidine
ICH: International Conference on Harmonization
*IFNs*: *InterFeroNs*
*IL-2*: *InterLeukin-2*
IRI: IRInotecan
IV: IntraVenously
KRAS: Kirsten RAt Sarcoma
LAK: Lymphokine Activated Killer
M1 and M2: Macrophages subclass 1 and subclass 2
mAb: monoclonal AntiBody
mCRC: metastatic ColoRectal Cancer
mg: milligram
NCCN: National Comprehensive Cancer Network
NK: Natural Killer
NRAS: Neuroblastoma Rat Sarcoma
OS: Overall Survival
PCR: Polymerase Chain Reaction
*PDGF*: *Platelet-Derived Growth Factor*
PFS: Progression-Free Survival
R: Arginine
RECIST: Response Evaluation Criteria In Solid Tumors
RR: Response Rate
SRB: SulfoRhodamine B
TAM: Tumor Associated Macrophages
*TGFb*: *Tumor Growth Factor b*
TIL: Tumor Infiltrating Lymphocites
TM: Tumor Microenvironment
V: Valine
*VEGF*: *Vascular Endothelial Growth Factor*
wt: wilde type

## References

[1] Herszényi L, Tulassay Z (2010). Epidemiology of gastrointestinal and liver tumors. Eur Rev Med Pharmacol Sci.

[2] Altekruse SF, Kosary CL, Krapcho M, Neyman N, Aminou R, Waldron W (2010). SEER cancer statistics review, 1975–2007.

[3] Lucas AS, O'Neil BH, Goldberg RM (2011). A decade of advances in cytotoxic chemotherapy for metastatic colorectal cancer. Clin Colorectal Cancer.

[4] Garrett CR, Eng C (2011). Cetuximab in the treatment of patients with colorectal cancer. Expert Opin Biol Ther.

[5] Mitchell RA, Luwor RB, Burgess AW (2018). Epidermal growth factor receptor: structure-function informing the design of anticancer therapeutics. Exp Cell Res.

[6] Zhao B, Wang L, Qiu H, Zhang M, Sun L, Peng P (2017). Mechanisms of resistance to anti-EGFR therapy in colorectal cancer. Oncotarget.

[7] Lo Nigro C, Ricci V, Vivenza D, Granetto C, Fabozzi T, Miraglio E (2016). Prognostic and predictive biomarkers in metastatic colorectal cancer anti-EGFR therapy. World J Gastroenterol.

[8] Denkert C, Darb-Esfahani S, Loibl S, Anagnostopoulos I, Jöhrens K (2011). Anti-cancer immune response mechanisms in neoadjuvant and targeted therapy. Semin Immunopathol.

[9] Ochoa MC, Minute L, Rodriguez I, Garasa S, Perez-Ruiz E, Inogés S (2017). Antibody-dependent cell cytotoxicity: immunotherapy strategies enhancing effector NK cells. Immunol Cell Biol.

[10] Mellor JD, Brown MP, Irving HR, Zalcberg JR, Dobrovic A (2013). A critical review of the role of fc gamma receptor polymorphisms in the response to monoclonal antibodies in cancer. J Hematol Oncol.

[11] Veluchamy JP, Spanholtz J, Tordoir M, Thijssen VL, Heideman DA, Verheul HM (2016). Combination of NK cells and Cetuximab to enhance anti-tumor responses in RAS mutant metastatic colorectal Cancer. PLoS One.

[12] Correale P, Marra M, Remondo C, Migali C, Misso G, Arcuri FP (2010). Cytotoxic drugs up-regulate epidermal growth factor receptor (EGFR) expression in colon cancer cells and enhance their susceptibility to EGFR-targeted antibody-dependent cell-mediated-cytotoxicity (ADCC). Eur J Cancer.

[13] Ochoa MC, Minute L, López A, Pérez-Ruiz E, Gomar C, Vasquez M (2017). Enhancement of antibody-dependent cellular cytotoxicity of cetuximab by a chimeric protein encompassing interleukin-15. Oncoimmunology.

[14] Cartron G, Dacheux L, Salles G, Solal-Celigny P, Bardos P, Colombat P (2002). Therapeutic activity of humanized anti-CD20 monoclonal antibody and polymorphism in IgG fc receptor FcγRIIIa gene. Blood.

[15] Weng WK, Levy R (2003). Two immunoglobulin G fragment C receptor polymorphisms independently predict response to rituximab in patients with follicular lymphoma. J Clin Oncol.

[16] Musolino A, Naldi N, Bortesi B, Pezzuolo D, Capelletti M, Missale G (2008). Immunoglobulin G fragment C receptor polymorphisms and clinical efficacy of trastuzumab-based therapy in patients with HER-2/neu-positive metastatic breast cancer. J Clin Oncol.

[17] Zhang W, Gordon M, Schultheis AM, Yang DY, Nagashima F, Azuma M (2007). FcγR2A and FcγR3A polymorphisms associated with clinical outcome of epidermal growth factor receptor–expressing metastatic colorectal cancer patients treated with single-agent Cetuximab. J Clin Oncol.

[18] Bibeau F, Lopez-Crapez E, Di Fiore F, Thezenas S, Ychou M, Blanchard F (2009). Impact of FcγRIIa-FcγRIIIa polymorphisms and KRAS mutations on the clinical outcome of patients with metastatic colorectal cancer treated with Cetuximab plus irinotecan. J Clin Oncol.

[19] Pander J, Heusinkveld M, van der Straaten T, Jordanova ES, Baak-Pablo R, Gelderblom H (2011). Activation of tumor-promoting type 2 macrophages by EGFR-targeting antibody Cetuximab. Clin Cancer Res.

[20] Calemma R, Ottaiano A, Trotta AM, Nasti G, Romano C, Napolitano M (2012). Fc gamma receptor IIIa polymorphisms in advanced colorectal cancer patients correlated with response to anti-EGFR antibodies and clinical outcome. J Transl Med.

[21] Trotta AM, Ottaiano A, Romano C, Nasti G, Nappi A, De Divitiis C (2016). Prospective evaluation of Cetuximab-mediated antibody-dependent cell cytotoxicity in metastatic colorectal Cancer patients predicts treatment efficacy. Cancer Immunol Res.

[22] Van Cutsem E, Köhne CH, Hitre E, Zaluski J, Chang Chien CR, Makhson A (2009). Cetuximab and chemotherapy as initial treatment for metastatic colorectal cancer. N Engl J Med.

[23] Heinemann V, von Weikersthal LF, Decker T, Kiani A, Vehling-Kaiser U, Al-Batran SE (2014). FOLFIRI plus cetuximab versus FOLFIRI plus bevacizumab as first-line treatment for patients with metastatic colorectal cancer (FIRE-3): a randomised, open-label, phase 3 trial. Lancet Oncol.

[24] Arnold D, Lueza B, Douillard JY, Peeters M, Lenz HJ, Venook A (2017). Prognostic and predictive value of primary tumour side in patients with RAS wild-type metastatic colorectal cancer treated with chemotherapy and EGFR directed antibodies in six randomized trials. Ann Oncol.

[25] Holch JW, Ricard I, Stintzing S, Modest DP, Heinemann V (2017). The relevance of primary tumour location in patients with metastatic colorectal cancer: a meta-analysis of first-line clinical trials. Eur J Cancer.

[26] Dienstmann R, Vermeulen L, Guinney J, Kopetz S, Tejpar S, Tabernero J (2017). Consensus molecular subtypes and the evolution of precision medicine in colorectal cancer. Nat Rev Cancer.

[27] Van Cutsem E, Cervantes A, Adam R, Sobrero A, Van Krieken JH, Aderka D (2016). ESMO consensus guidelines for the management of patients with metastatic colorectal cancer. Ann Oncol.

[28] Eisenhauer EA, Therasse P, Bogaerts J, Schwartz LH, Sargent D, Ford R (2009). New response evaluation criteria in solid tumours: Revised RECIST guideline (version 1.1). Eur J Cancer.

[29] Simon R (1989). Optimal two-stage designs for phase II clinical trials. Control Clin Trials.

[30] Lacouture ME, Anadkat M, Jatoi A, Garawin T, Bohac C, Mitchell E (2018). Dermatologic toxicity occurring during anti-EGFR monoclonal inhibitor therapy in patients with metastatic colorectal Cancer: a systematic review. Clin Colorectal Cancer.

[31] Papazisis KT, Geromichalos GD, Dimitriadis KA, Kortsaris AH (1997). Optimization of the sulforhodamine B colorimetric assay. J Immunol Methods.

[32] Skehan P, Storeng R, Scudiero D, Monks A, McMahon J, Vistica D (1990). New colorimetric cytotoxicity assay for anticancer-drug screening. J Natl Cancer.

[33] Studzinski GP, Skehan P, Studzinski GP (1995). Assays of cell growth and cytotoxicity. Cell growth and apoptosis: a practical approach.

[34] Tol J, Punt CJ (2010). Monoclonal antibodies in the treatment of metastatic colorectal cancer: a review. Clin Ther.

[35] Bardelli A, Siena S (2010). Molecular mechanisms of resistance to cetuximab and panitumumab in colorectal cancer. J Clin Oncol.

[36] Van Cutsem E, Tejpar S, Vanbeckevoort D, Peeters M, Humblet Y, Gelderblom H (2012). Intrapatient cetuximab dose escalation in metastatic colorectal cancer according to the grade of early skin reactions: the randomized EVEREST study. J Clin Oncol.

[37] Overdijk MB, Verploegen S, van den Brakel JH, Lammerts van Bueren JJ, Vink T, van de Winkel JG (2011). Epidermal growth factor receptor (EGFR) antibody-induced antibody-dependent cellular cytotoxicity plays a prominent role in inhibiting tumorigenesis, even of tumor cells insensitive to EGFR signaling inhibition. J Immunol.

[38] Seo Y, Ishii Y, Ochiai H, Fukuda K, Akimoto S, Hayashida T (2014). Cetuximab-mediated ADCC activity is correlated with the cell surface expression level of EGFR but not with the KRAS/BRAF mutational status in colorectal cancer. Oncol Rep.

[39] Church SE, Galon J (2017). Regulation of CTL infiltration within the tumor microenvironment. Adv Exp Med Biol.

[40] van Pelt GW, Sandberg TP, Morreau H, Gelderblom H, van Krieken JHJM, Tollenaar RAEM (2018). The tumour-stroma ratio in colon cancer: the biological role and its prognostic impact. Histopathology.

[41] Zhong X, Chen B, Yang Z (2018). The role of tumor-associated macrophages in colorectal carcinoma progression. Cell Physiol Biochem.

[42] Franklin RA, Liao W, Sarkar A, Kim MV, Bivona MR, Liu K (2014). The cellular and molecular origin of tumor-associated macrophages. Science.

[43] Vlaicu P, Mertins P, Mayr T, Widschwendter P, Ataseven B, Hogel B (2013). Monocytes/macrophages support mammary tumor invasivity by co-secreting lineage-specific EGFR ligands and a STAT3 activator. BMC Cancer.

[44] Wyckoff JB, Wang Y, Lin EY, Li JF, Goswami S, Stanley ER (2007). Direct visualization of macrophage-assisted tumor cell intravasation in mammary tumors. Cancer Res.

[45] Krneta T, Gillgrass A, Poznanski S, Chew M, Lee AJ, Kolb M (2017). M2-polarized and tumor-associated macrophages alter NK cell phenotype and function in a contact-dependent manner. J Leukoc Biol.

[46] Luo Y, Zhou H, Krueger J, Kaplan C, Lee SH, Dolman C (2006). Targeting tumor-associated macrophages as a novel strategy against breast cancer. J Clin Invest.

[47] O’Sullivan T, Saddawi-Konefka R, Vermi W, Koebel CM, Arthur C, White JM (2012). Cancer immunoediting by the innate immune system in the absence of adaptive immunity. J Exp Med.

[48] Muntasell A, Ochoa MC, Cordeiro L, Berraondo P (2017). López-Díaz de Cerio a, Cabo M, et al. targeting NK-cell checkpoints for cancer immunotherapy. Curr Opin Immunol.

